# Development of a psycho-social intervention for reducing psychological distress among parents of children with intellectual disabilities in Malawi

**DOI:** 10.1371/journal.pone.0210855

**Published:** 2019-02-11

**Authors:** Charles Masulani-Mwale, Felix Kauye, Melissa Gladstone, Don Mathanga

**Affiliations:** 1 St. John of God Mental Health Services, Malawi, Mzuzu, Malawi; 2 Department of Community Health, University of Malawi College of Medicine, Blantyre, Malawi; 3 University of Liverpool, UK Department of Women and Children’s Health, Institute of Translational Medicine, University of Liverpool, Alder Hey Children’s NHS Foundation Trust, Liverpool, United Kingdom; Università degli Studi di Perugia, ITALY

## Abstract

**Background:**

The burden of intellectual disabilities in low and middle income countries (LMIC) is high and is associated with parental psychological distress. There are few services for children and parents in most developing countries and few interventions have been created that target the psychological issues among parents of such children. This study aimed to develop a contextualized intervention to provide psychological support for parents of children with intellectual disabilities in an African setting.

**Methods:**

Six steps were adopted from the Medical Research Council framework for designing complex interventions. This include: literature review of similar interventions and models, qualitative studies to gain insights of lived experiences of parents of such children, a consensus process with an expert panel of professionals working with children with disabilities and piloting and pre-testing the draft intervention for its acceptability and practicability in this settings.

**Results:**

21 intervention modules were found from a systematic search of the literature which were listed for possible use in our intervention along with four themes from our qualitative studies. An expert panel formed consensus on the eight most pertinent and relevant modules for our setting. This formed the intervention; “Titukulane.” This intervention was piloted and found to have high acceptability and practicability when contextualized in the field.

**Conclusion:**

The use of a systematic framework for designing a complex intervention for supporting the mental health of parents of children with disabilities enables good acceptability and practicability for future use in low resource settings.

## Introduction

Intellectual disabilities place a high burden of disease on low and middle income countries [LMIC) and are a public health priority because of their continuation through the life course and impact on families [1]. Despite this, there are very few services or interventions for these children and their families in LMIC settings, particularly for those of preschool age [2]. Parents of children with intellectual disabilities can have negative experiences in caring for their children and may need specific services for their children as well as psychological interventions for their own mental health issues [3, 4].

While psychological interventions for parents of children with disabilities have been advanced in the developed world [5, 6], there is a sparsity in African settings where parents may be even more needy [4, 7, 8]. Very few psychological interventions in Africa have been tested or demonstrated positive effects in reducing psychological distress. Those which have been tested, include general psychological interventions for adults, in Zimbabwe and in South Asia [9–12]. No studies have studied the effect psychological interventions with parents, particularly those with children with intellectual disabilities.

When considering interventions that may work in African settings, an intervention with proven efficacy in high income countries (HIC) could be used. This strategy however, may lead to lack of validity in settings that are culturally very different. Secondly, interventions from HIC are often specialized and performed by highly skilled professionals. Administration can be expensive and with the scarcity of financial resources in low income countries, this raises questions of feasibility and sustainability in implementing these interventions [13].

In order to target the treatment gap for psychosocial and mental health care for parents of children with intellectual disabilities in low and middle income countries, there are numerous barriers which need to be addressed in order to scale up care in these settings. This needs to start with studies which look at feasibility and acceptability of culturally appropriate interventions for specific settings. In this study, we aim to address this gap by designing an intervention in a systematic and stepwise manner which is shown to be feasible and acceptable in our setting. Once we demonstrate feasibility and acceptability, we will be more able to scale up the intervention and understand whether it may be effective in reducing psychological problems among parents of children with intellectual disability in Africa.

### Study aims

The overall purpose of this study is to develop an intervention/programme which is acceptable and feasible to parents and which will provide psychological support for parents of children with intellectual disabilities using an evidence based approach. The programme will be for use in Malawi where there is limited access to community rehabilitation or mental health and allied support services. The aim of the training resource is to empower parents, families, and communities with increased knowledge about intellectual disability, and to reduce stigma and myths associated with disability in order to reduce associated negative mental health outcomes.

The specific objectives of the study were as follows; (a) to utilize a literature review to create a theory of how psychological interventions help to alleviate psychosocial problems in parents of children with disabilities and to analyze and choose effective theories and interventions which might work in this setting, (b) to utilize qualitative information gathered in qualitative themes gathered in studies with parents of children with disabilities in order to inform us of the pertinent issues to address [14] relating to the psychological needs of parents (c) to undertake a consensus process with an expert panel of professionals to identify which themes and subjects would be most effective to address in a Malawian setting and (d) to understand through piloting and pre-testing the practicability, the ability to recruit and retain participants and the participants perception regarding timings of the programme and acceptability this intervention by the parents.

## Methods

This study received ethical approval from the Malawi College of Medicine Research and Ethics Committee (# P.06/14/1591) as well as from the medical director of Likuni where the pilot study was done. Written informed consent was obtained from the participants before inclusion into the study by explaining nature of the study (about the procedure, its purpose, being assured of confidentiality of the information and risks and benefits). All ethical issues and anonymity of the identity of participation as well as the rights of their children were respected during the study process.

Consent was also sought from participants for publishing the findings of the study in peer reviewed journals

We used the Medical Research Council (MRC) framework for developing and evaluating (non-pharmacological) interventions [15] in order to follow a stepwise rigorous framework to enable us to create our intervention (referred here in as “Titukulane” a local vernacular name meaning “Lets empower each other”) in an evidence based fashion. After reviewing various intervention development procedures, six stages were adapted in the development of this intervention.

### Titukulane development process

**Fig 1:**
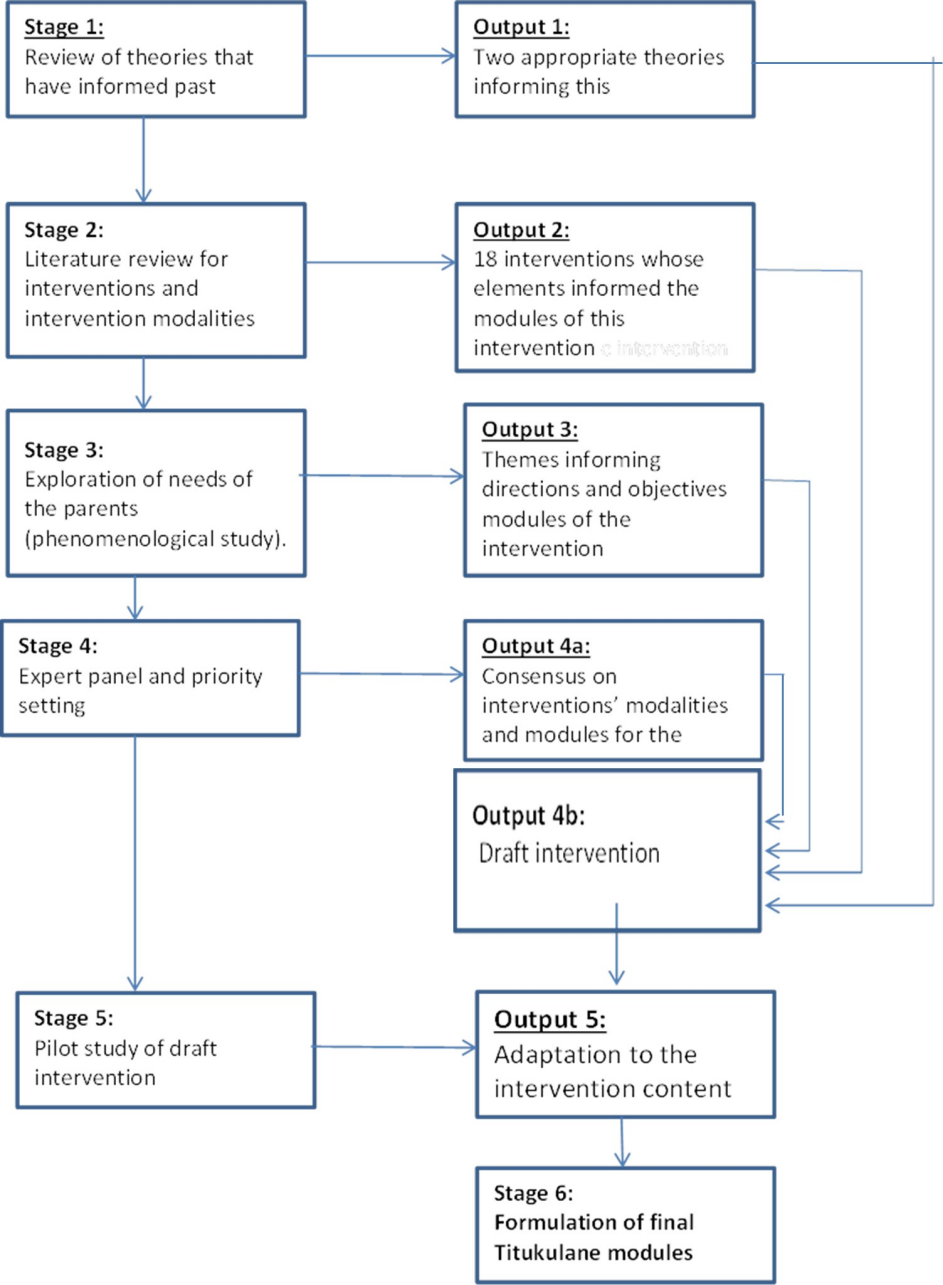
Titukulane intervention development process showing stages and output at each stage.

The intervention development process that was followed in this study consisted of six stages listed below. (Fig 1)

### Study setting

Our studies took place in the Western and Northern part of Mzuzu and Lilongwe cities respectively.

#### Stage 1: Identification of theories relating to intervention development which are likely to fit into Malawian context

This stage involved determining the appropriate theories which would link with the correct intervention development plan. We utilized our review of the literature [16] to identify two models; the “Family Care Model” and the “Risk Resilient Model” which we considered as the most relevant theorised frameworks for our intervention development process.

The Family Care Model provides a model which promotes an understanding of the interaction of parenting a disabled child and parental mental health. This model uses the theory of stress and coping theory and identifies three major stages [17, 18] including the child and caregiver or family characteristics; mediators and parental perceptions and coping behaviours–all which play an influence on caregiver stress.

The Risk Resilient model [19] considers risk factors (parameters of child’s disability, carers’ functional care strain and carers’ psychosocial stress) and resistance or resilient factors of the carers that alleviate the stressors (coping, supports and social-ecological factors). As our proposed intervention (parent training) promotes parents’ coping skills with the aim to reduce psychological distress and promote psychological quality of life, both these models were utilised in the expert panel’s prioritisation of topics in subsequent stages.

#### Stage 2: Literature review of previous developed interventions’ and their modalities

We reviewed literature for evidence-based interventions for parents of children with disabilities in order to subsequently distil a profile of common modules or practices among reviewed efficacious interventions from LMIC settings. We aimed to identify Interventions that had a track-record in LMIC countries but due to scarcity of these in LMICs, those from high income countries were also considered. We detail our review in another publication [4].

#### Stage 3: Phenomenological qualitative study to identify themes relating to psychological experiences and needs of parents of children with disabilities

We conducted a qualitative study, aimed at exploring the psychological experiences of parents caring for children with intellectual disabilities. We aimed to understand their mechanisms of coping with these challenges and their psychosocial needs in two rural areas surrounding Mzuzu and Lilongwe. We identified themes from this phenomenological study which were then considered in stage 4. The results of this study are provided in another paper [14].

#### Stage 4: Expert panel review and priority rating of intervention topics

In this stage, an expert panel, comprising the first author (CM), two expert psychologists (NS, WN) and two highly experienced psychiatric officers (LN and VS), was assembled to list and rank the generated intervention options. These experts were identified based on their wealth of experience in working with parents and children with disabilities in Malawi. The expert panel used guidelines to systematically set priorities in health research investments [20, 21]. This included; (a) gathering the five experts in four center-based face-to-face meetings (b) systematically listing the intervention options/modules from the interventions/packages that were identified in the literature. Thirty common modules identified in this process are as listed in Table 1 below and (c) scoring listed options by pre-defined criteria which includes acceptability, feasibility and effectiveness as recommended by CHNRI and outlined in Table 1 [20, 21].

**Table 1:** Pre-determined criterion questions used by the expert panel as recommended by CHNRI.

CRITERIA	EXPLANATION OF CRITERIA
**Criterion 1**: assessment of the cultural and ethical **acceptability** of the intervention	1. Taking into account the socio-cultural context (including care belief systems) wherein the intervention is to be used, would you say that the intervention is culturally acceptable? 2. Taking into account that mental health intervention might harm beneficiaries, would you say that this intervention safeguard and protect its recipients
***Criterion 2***: assessment of **feasibility** (deliverability and affordability) of the intervention	1. Taking into account the resources available to implement the intervention, would you say that the intervention is affordable in a low-income setting? 2. Taking into account the level of difficulty with intervention delivery (i.e. standardizability, required human resources, community infrastructure), would you say that the intervention would be deliverable within the context of interest?
***Criterion 3***: assessment of likelihood that the intervention would be **effective**	1. Based on the best existing evidence and knowledge, would the intervention that would be utilized be effective?

In prioritizing the 30 items, each of the panelists ranked each item against three criteria (Table 1) using categories of; “strongly agree” = 2; “agree” = 1; “disagree” = 0 or “insufficiently informed to answer” = blank. The highest possible score on each item was 3. For all five panelist, the highest possible score on each was therefore 30. The final outcome was to have between eight to ten modules from the table to be used in the intervention. After adding the total scores for each of the five panelists, all items that had a total score of 25 and above were prioritized for inclusion in the intervention.

#### Stage 5: Qualitative assessment of acceptability and feasibility of the tool of newly created psychosocial intervention

The fifth step was a qualitative assessment of the draft intervention through a pilot study using focus group discussions and consequently making adaptations to the intervention content or modality to improve feasibility and cultural acceptability. This part used a qualitative phenomenological design to explore the lived experiences of parents of children with disabilities who had undergone the pilot study of the intervention. Fourteen mothers and fathers of children with intellectual disabilities, aged from 18 and above, were purposively sampled from the intervention sites after the intervention. We conducted two focus group discussions (FGDs)—one for 6 males and one for 8 females in September 2015. The FGDs and interviews were held in private rooms. A topic guide was created with extra probes which included questions such as; What they learnt? What they found helpful? What could be done to improve the facilitation strategies? What part of contents of the training could they retain after one month? All interviews were tape-recorded, transcribed, and translated from Chichewa to English by the investigator (CM). A thematic analysis of the transcripts was adopted to understand the data. During coding, major nodes were first created; patterns were identified and put together to form tree nodes each bearing a name of a sub theme. Word-for-word quotes from the transcripts are reported in the findings below.

## Results

### Stage 1: Identification of models relating to intervention development which are likely to fit in a Malawian context

While some elements from the Risk Resilient model were considered because of its emphasis on risk factors, the Family Care Model was identified as the most appropriate model for development of this particular intervention. This was because it provides an easy understanding of the interaction of parenting a disabled child and parental mental health.

### Stage 2: Literature review of previous developed interventions’ and their modalities

Our search of literature found 21 full-text articles after a search of over 1000 articles. The interventions were selected and used for this review were those that had relevance to informing future policy, research and clinical practice. Some of the interventions reviewed included: Sign Posts training [6], Hambisela [22], Incredible years parents’ training [5], Stepping stone Triple- P[23, 24], Problem-Solving Education [PSE] [25], Behavioral Parent Training Program [26], Mindfulness-based Stress Reduction [27], Parent-Child-Interaction Therapy [PCIT] [28], Augmentative and Alternative communication [29, 30] and Portage [31]. The common modules identified are listed in Table 2.

**Table 2:** Modules as prioritized during the expert panel excise.

Module	Acceptability	Feasibility	Effectiveness	Total score
**1. Introduction to disability**	9	9	10	28*
**2. Measuring your child’s behavior**	2	3	5	10
**3. Systematic use of everyday interactions**	8	10	7	25
**4. Replacing difficult behavior with useful behavior**	3	2	4	9
**5. Teaching your child new skills**	6	5	7	18
**6. Dealing with stress and other mental health issues**	10	10	10	30*
**7. Your famil*y* as a team.**	4	6	5	15
**8. Following Human development**	10	7	10	27*
**9. Respect and understanding children and their developmental abilities, modelling social skills, child‐directed play, balancing power**	7	6	8	21
**10. Having developmentally appropriate expectations for child**	5	8	7	20
**11. Positive parenting, controlling emotions and improving relationships, effective communication skills, family problem solving, enhancing children’s learning, anger management, and managing conflict.**	8	8	9	25
**12. Counselling for carers of disabled children**	8	10	10	28*
**13. Establishing rules, predictable routines**	4	7	5	16
**14. Increasing the understanding of cerebral palsy as a condition, and how it affects people.**	10	9	10	29*
**15. Increase the level of basic skills in handling, feeding, communication, play, and everyday activities.**	8	7	8	23
**16. Roles of professional and caregivers**	10	10	10	30*
**17. Positioning and Carrying a child**	8	8	8	24
**18. Communication**	7	8	6	21
**19. Feeding your Child**	4	5	7	16
**20. Playing with your child**	5	6	4	15
**21. Disability in your Local Community.**	8	8	8	24
**22. Legal protection of disabled children**	10	10	10	30*
**23. Running your Own Parent Support Group**	9	8	8	25
**24. Using parental attention to change behavior**	4	6	5	15
**25. Encouragement and praise**	4	5	7	16
**26. Using reward systems effectively**	9	8	9	26
**27. How to set rules and handle misbehavior & How to set rules and help children keep them**	8	7	9	24
**28. How to use active ignoring to reduce misbehavior**	5	7	4	16
**29. Using time out and other sanctions**	4	3	6	13
**30. Managing psychological issues among parents**	10	10	10	30*

* Indicates modules that had total scores above 25 in the Table 2 and were retained in the intervention manual.

### Stage 3: A phenomenological qualitative study to identify themes relating to psychological experiences and needs of parents of children with disabilities

The themes identified from the qualitative studies [32] included: child challenging behaviors: community perception and stigma; marital issues; service shopping; worries; unmanaged stress and depression; fears and worries for the future; suicide; filicide; positive coping; spirituality; poor coping mechanism; material needs; counseling; and respite services. These themes are included in the first column of Table 2 which outlines results of scoring of the modules identified from literature review as well as the themes from our qualitative studies.

### Stage 4: Expert panel review and priority rating of intervention topics

The scores from the expert panel are shown in Table 2. Those with total scores above 25 are *starred in Table 2 and were retained in our intervention manual.

### Stage 5: Acceptability and feasibility of Titukulane

Four themes emerged from our qualitative including: “Content of the material learned”, “Perceived usefulness of the training”, “Areas suggested for improvement” and “Subject content and memory retention period.”

#### Theme 1. Content of the training materials

Most parents enjoyed the training and were able to describe what they had learnt during the training. Many parents found the training very holistic addressing issues such as general health and mental health issues for children; alleviation of poverty through business ventures; psychological support such as “managing your thoughts” and even about general child care practices. Examples of the what parents enjoyed are demonstrated below:

*“We were told about stages of child development and how we can care for our children; how we can stop stigmatizing them nor torturing them so that they can be happy and grow well”* (FGD1, LK, women).

*“There were many lessons but I vividly remember that we were told that there are three components of a person including*: *spirit*, *body and mind… the also helped us on how we can start a business and make profits*.*”* (FGD2, LK, Men).

*“…yes it was very good and* ..*they also talked about how we can stabilize our thoughts and where we can get help”* (FGD1, LK, women).

#### Theme 2. Perceived usefulness of the training

Parents were asked what they found most useful during the training. Many cited how the training helped them to understand explanations for their un-explained medical complaints and how to deal with distressing moments;

*“… fears are now gone…*..*we learned a lot…*. *that some headaches are due to thoughts” (FGD2*, *LK*, *Men)*.

*“…I did not understand what was happening in my body before that training*. *I had general body weaknesses and…no appetite*. *During the sessions we were told that thoughts and worries eat us and make us sick too*. *After…*..*training*, *we now know how to control ourselves even to avoid thinking of killing self*.*” (FGD1*, *LK*, *Women)*.

One lady also said that, *“I used to cry daily thinking about my child’s issues and that he cannot even go to school*… . *it was painful but the training helped me to have hope and restart life again*.*”*

The parents mentioned how the training had helped them gain insights into the causes of their child’s disability and clarify some societal myths associated with having a disabled child. *“There were a lot of things people were talking about us including that the child’s disability was caused by our unfaithfulness in the family*. *This training gave us the various factors that can case disability not the hearsays we used to know”*. *(FGD2*, *LK*, *Men)*.

Adding to this, some described how they had started socializing and even resumed sexual activities with spouses; *“In our family we now can enjoy sex than before whereby we feared getting pregnant and having another child with the same problem”*. *(FGD1*, *LK*, *Women)*.

Some parents also mentioned that the training helped to learn how to care for their children better*; “I learnt how to properly and easily feed my child and how to position him*, *all because of the training*.*” (FGD1*, *LK*, *Women)*.

#### Theme 3. Areas suggested for improvement

Parents were asked what they felt could be done to improve the training. In general, participants were very happy with the content and facilitation of the training but wanted more of it; *“The training was done very well but it needs to be done frequently*” (FGD1, LK, Women) with transport to and from sessions*; “*..*everything went well*, *the facilitators were excellent however time was two short…*.. *next time provide transport to allow all parents to come with their children so that they can also know their friends*” (FGD1, LK, Women). One participant requested that a school be built so that their children can learn some skills and be cared for in a separate environment.

Some participants also mentioned the need to support participant with transport money to reduce absenteeism due lack of transport money to the training venue.

#### Theme 4. Subject content and memory retention period

Participants were also asked about how long they felt they could remember different modules covered in the training. Most said they could not forget most of the material covered; “*We can’t forget the information that we were given*, *it is settled in our minds*.*” (FGD2*, *LK*, *Men)*.

### Analysis of themes and proposed Titukulane modules

We synchronized the themes from the qualitative study in stage 3 and the prioritized modules in stage 4 to make a final decision about which modules to include in the final version of the training programme. The Table 3 below shows the qualitative study themes and corresponding modules of the Titukulane.

**Table 3:** Themes and corresponding modules of Titukulane.

Study themes		
Main themes	Subthemes	
1. Challenges in Care	Marital issues	Module 3a-3b
	Community perception and stigma	Module 3b-4a
	Child care challenges	Module 1a &2a
2. *Mental issues*	*Worries*	Module 3a-3b
	*Stress and depression*	Module 3a-3b
	*Fears for the future*	Module 3a-3b
	*Suicide/ Infanticide/ Filicide*	Module 3a-3b
3. *Coping mechanisms*	*Positive coping*	Module 3a-3b
	*Poor coping*	Module 3a-3b
4. *Support needs*	*Respite services*	Module ??
	*Education about disability*	Module 1a-2a
	*Counseling*	Module 3b
	*Material needs*	Module 4a

### Further changes to intervention

The Titukulane delivery period was modified to address needs of parents to make the initial draft manual more feasible and acceptable for use. After further analysis of issues and themes highlighted by parents, most issues were found to be addressed by different components of the modules already provided except for; financial and material needs (poverty) issues and need for respite services.

To address the issues of poverty, the Self-help groups’ module [33] (a financial empowerment approach) was adopted from the St John of God services to address the poverty issues through promotion of savings culture and small business enterprises. The issue relating to respite services was not included in our package as there is a current advocacy for community based disability care and that respite care is too complex and systematized.

### Stage 6: Final creation of the Titukulane intervention

The final result is a community-accepted package, “Titukulane” which we have based on review of efficacious interventions, expert opinion of modalities, formative qualitative studies as well as later qualitative feasibility and acceptability studies which has now been pilot-tested in Malawi. It contains the following modules:

- Module 1a: Introduction on the human development
- Module 1b: Intellectual disability and types of Intellectual Disability
- Module 2a: Causes, prevention and management of children with Intellectual Disability
- Module 2b: Roles and responsibilities of caregivers of children with Intellectual Disability
- Module 3a: Common mental health issues among carers of children with Intellectual Disability
- Module 3b: Group Counselling, Parenting and coping with common mental health issues
- 4a: Self-help groups and other available support systems
- 4b: Rights for children with intellectual disability and their carers.

## Discussion

We have created one of the first interventions for training parents of children with intellectual disabilities which has gone through a rigorous process for development of the intervention for a low income African setting. The Titikulane intervention is based on modules that were found through literature review to be most effective as well as modules created through our formative qualitative work. The modules felt to be most acceptable, feasible and effective were chosen by an expert panel, piloted and then reviewed along with further qualitative data from parents and caregivers. The theoretical basis of this Titukulane derived from Family Care Model. This model provides an easy understanding of the interaction of parenting a disabled child and parental mental health and was previously built on positive outcomes from group based interventions. We hoped that by using this model, an intervention designed to help parents to cope with the challenging roles would results in reduction of mental health problems among the parents. [34]. By connecting theory to the intervention development, we feel that we have come up with a programme which will more be able to facilitate coping among the parents of children with intellectual disabilities in a way we know works theoretically in other settings.

Our previous qualitative studies demonstrated that parents hardly access services for their children, let alone for their own psychological issues; they experience stigma and discrimination; have unmanaged psychological stress; have self-blame and guilt; have suicidal ideas and in some cases have even been coerced by neighbors to kill their disabled child. Their needs include; respite, improved access to disability services, education on disability management and financial support. For emotional stability, some parents cope by sharing with others and through their spirituality while some have poor coping mechanisms. This echoes with previous work demonstrating that care of children with disabilities impact negatively among parents especially mothers [35, 36]. While there were varied coping mechanism among the parents, spirituality and its effect on coping was a major theme within our study. This indicates that culturally acceptable and evidence based programs should be designed, for low income settings, to nurture positive attitudes, skills, and knowledge.

The results from our feasibility and acceptability work conducted as part of the pilot intervention carried out in this study shows that this Titukulane is perceived to be feasible and useful by parents of intellectually disabled children. This work mirrors similar work in Pakistan where a parent-based community intervention was created to be delivered by Lady Health Workers as part of their routine duties, in a poor rural area of Pakistan, for children with mental retardation [37]. While our study has not been triangulated and was carried out on a small sample of parents in two cities, we are confident that the results can be generalizable. Our intervention has been developed after careful systematic review and expert panel prioritization of the modules in consideration to the psychological needs of the parents. While results from this and our related studies have informed us about the psychological burden among parents [32], our pilot study does not inform us about the program’s impact on child outcomes such as behavior and functioning. For future work, it will be important to triangulate our study by measuring quantitative outcomes such as maternal stress, family functioning and quality of life and child behavior and functioning over a longer period of time to assess if this intervention is, indeed, effective. Furthermore, during this pilot study we have found that those who drop out are the most vulnerable and often need extra support at home. We therefore suggest that for future versions of our programme, it will be important to follow up with those who terminate the training, to understand their reasons and how to best support them.

Prior to this study, in low income settings, only non-randomised studies have been used to reach conclusions [38]. Considering the socio-economic differences between high and low income countries, there could be multiple challenges in developing and implementing such interventions. These include lack of personnel and expertise to facilitate training programs; limited resources to use for the interventions; limited access by the potential participants due to transportation problems; and contextual differences affecting cultural acceptability of the intervention. It is therefore vital that interventions are adapted to fit within the local cultural context and are acceptable for parents in these settings if we want to promote mental health resilience. With limited resources and fewer psychologists, low-income countries need to have the evidence that the cost of providing interventions particularly when provided through lower skilled community workers are beneficial to the psychosocial outcomes of parents in these settings. We have done this within this present study and hope that we have now made a programme that is culturally relevant and strong in terms of its theoretical and evidence based underpinnings. The intervention which we have now developed now needs to be trialed to prove its effectiveness in reducing psychological distress among parents.

### Study strengths and limitations

While this study is the only one to design an intervention with much rigor in Central Africa, it has some limitations. The study comprised of parents of children with intellectual disability and therefore the views of these parents may differ from those of parents with children with other forms of disability. Although many children of caregivers in the study had mixed and complex disabilities, it may be that those which were excluded, for example those with pure visual or hearing impairment or those with physical disabilities. These children may cause less stress than those with intellectual disabilities but it is very difficult to know this without doing further quantitative research. In short, a generalization of these findings may be limited to parents attending CBR clinics due to non-inclusion of parents whose children have never been to any clinics.

### Implication for future research and practice

Future research using a randomized controlled design and a large and representative sample would be appropriate to test these outcomes over the long term among parents and their children. The major strength of this community based group intervention is that it can ensure maximum population coverage, with an emphasis on solving problems that are common and prioritized by the parents. The intervention, also, had a component of economic empowerment which can help alleviate the issues of poverty highlighted by many parents.
